# Challenges to Achieving Sustainable Sanitation in Informal Settlements of Kigali, Rwanda

**DOI:** 10.3390/ijerph10126939

**Published:** 2013-12-10

**Authors:** Aime Tsinda, Pamela Abbott, Steve Pedley, Katrina Charles, Jane Adogo, Kenan Okurut, Jonathan Chenoweth

**Affiliations:** 1 Centre for Environmental Strategy, Faculty of Engineering and Physical Sciences, University of Surrey, Guildford GU2 7XH, UK; E-Mails: k.charles@surrey.ac.uk (K.C.); k.okurut@surrey.ac.uk (K.O.); j.chenoweth@surrey.ac.uk (J.C.); 2 Institute of Policy Analysis and Research, IPAR-Rwanda, Kigali 273, Rwanda; E-Mail: p.abbott@abdn.ac.uk; 3 Robens Centre for Public and Environmental Health, University of Surrey, Guildford GU27XH, UK; E-Mail: s.pedley@surrey.ac.uk; 4 School of Law, Faculty of Business, Economics and Law, University of Surrey, Guildford, Surrey GU2 7XH, UK; E-Mail: j.adogo@surrey.ac.uk

**Keywords:** challenges, issues, sustainable sanitation systems, informal settlements, Kigali

## Abstract

Like most cities in developing countries, Kigali is experiencing rapid urbanisation leading to an increase in the urban population and rapid growth in the size and number of informal settlements. More than 60% of the city’s population resides in these settlements, where they experience inadequate and poor quality urban services including sanitation. This article discusses the issues and constraints related to the provision of sustainable sanitation in the informal settlements in Kigali. Two informal settlements (Gatsata and Kimisagara) were selected for the study, which used a mixed method approach for data collection. The research found that residents experienced multiple problems because of poor sanitation and that the main barrier to improved sanitation was cost. Findings from this study can be used by the city authorities in the planning of effective sanitation intervention strategies for communities in informal settlements.

## 1. Introduction

In Kigali, Rwanda’s capital city, like many other cities in developing countries, the most widely used sanitary facilities in the poor neighbourhoods are pit latrines, occasionally supplemented with flushing toilets and septic tanks [1]. Conventional pit latrines provide a cheap way to handle human waste and require little maintenance; however, they provide limited comfort, attract flies and spread diseases such as diarrhoea and dysentery through contamination of the environment [2]. 

Rapid population growth and urbanization associated with the proliferation of informal settlements are often accompanied by environmental degradation. About 62% of the urban population in sub-Saharan Africa lives in informal settlements [3,4]. The problem of informal settlements remains one of the greatest challenges for city managers. In sub-Saharan Africa, less than half of the population has access to a sanitation facility that meets the WHO/UNICEF Joint Monitoring Program (JMP) definition of improved [5,6].

In Kigali, the population is growing faster than the provision of services. In 1996, the population was 358,200, but by 2012, it had increased to 1,135,428 [7]. Much of the urban growth has taken place in unplanned settlements that now accommodate 62.6% of the population. The 2010 Demographic and Health Survey reported 88.7% of sanitation to be improved; although this number falls to 46.2% if the JMP definition, which excludes shared sanitation, is used [8]. However, this percentage does not point out the disparities in conditions within the formal and informal parts of the urban area. 

Pit latrines in the informal settlements are often poorly maintained and rarely emptied; the pits are generally not lined with bricks and can collapse after a period of use [9]. Furthermore, there are few suction trucks available to empty soakage pits and septic tanks, and often sites are not accessible due to the narrow steep roads which lead to the latrines. Even if there is a possibility to empty liquid from pits, the sludge is not always disposed of in a proper manner. However, neglecting pit emptying or employing poor quality emptying services can have serious health and environment consequences. For example, substandard pit emptying services in Freetown, Sierra Leone, have been partly responsible for diarrhoeal disease, cholera outbreaks and high infant mortality, especially in informal settlements and poor unplanned areas [10,11]. 

This situation might be improved if Kigali was equipped with a sewerage system. However, unlike other cities in East Africa, which have networks of sewer pipes and treatment plants to cover a small percentage of its inhabitants, Kigali has neither a central treatment facility for sewage nor a system of sewers. As a result, the large volumes of wastewater produced in the city, are either discharged untreated into wetlands surrounding the city or allowed to infiltrate into ground water, polluting fresh water resources as well as the soil [1]. There are plans to build a sewage treatment plant, but funding remains a major challenge. Kigali lacks financial resources and therefore cannot afford a centralized sanitation system because of the high cost of the associated physical infrastructure which includes a network of pipes, treatment plants and maintenance [1,12]. 

Although it is known that well-managed systems for piped water, sanitation, drainage, and garbage removal would improve the health of city residents [13], introducing and maintaining centralised systems in developing cities have been hampered by political, economic, ecological and social instabilities. This leads to poor environmental performances and perpetual breakdowns, due to lack of proper maintenance or timely investments [2]. In addition, although Kigali is well-known for the cleanliness of its streets, little is known about the life in informal settlements. 

This raises a pressing need to understand the nature and magnitude of the issues affecting sanitation provision in order to find more cost-efficient and sustainable sanitation alternatives to address them. Innovative decentralized sanitation and re-use systems were developed partly in opposition to centralized ones and there have been claims they are more robust, cheaper and better able to deal effectively with environmental challenges [1]. Whichever technologies are used, they must be context appropriate and cost effective to the low-income dwellers [14] of developing cities. However, to the best of our knowledge, no research has been undertaken on how current on-site problems can be solved by the use of other on-site or ‘mixed’ technologies that match with the context of informal settlements of Kigali.

It is against this background that this article aims to analyse the challenges faced by people using the existing sanitation systems in terms of wastewater treatment, operation and maintenance; and frame sustainable sanitation systems that match with the local conditions of informal settlements of Gatsata and Kimisagara. The findings will contribute to providing the basis for policy makers to make informed decisions on which sanitation systems fit for informal settlements of Kigali, and other major cities. 

## 2. Methods

The study was conducted in two informal settlements in Kigali which were purposely sampled because they have some of the poorest sanitation facilities. The settlements are also characterized by high levels of poverty, high rates of illiteracy, high unemployment, poor housing, and a lack of access to quality health care and transportation, and an unhealthy environment [15]. Other characteristics of these areas include poor drainage systems, poor sanitation facilities, the unauthorized building of extensions, and the high density of settlements with steep slopes and wetland areas. 

A mixed method approach was used. This included transect walks through the settlements, a household survey, focus groups discussions and key informant interviews. The study team started an unannounced transect walk with an informal talk with a few community members and then continued the walk to observe the condition of sanitation facilities as well as any evidence of open defecation around the house and backyards. 

The household survey, collected quantitative data on sanitation facilities and income using a structured questionnaire. The survey sample was selected through random route sampling techniques in proportion to the population of the study area. It was conducted between May and September 2012. The survey questionnaire was pilot tested before being administered in the communities, and all the staff involved with the survey were trained before use. Out of the 1,883 targeted household, 1,794 households (95%) were interviewed giving a non-response rate of 5%. The head of household or another adult (18 years and over) answered the questionnaire on behalf of the household.

The findings of the survey were supplemented by the qualitative research, undertaken to find out more about the issues and constraints to improving sustainable sanitation. Purposive sampling was used to select informants with the same sampling frame used in both settlements. Focus group discussions and in-depth interviews were used to capture the informants’ perspective and allowed for more in-depth information on sanitation and helped us to better understand what was going on and why. This is important because in various fields of science, voices have been raised that research should be done with people and not on or for people [16,17]. Sanitation is an example for such a setting and thus in order to define sanitation technology options with a high chance of long-term success, a thorough understanding of the needs and concerns of residents (own occupiers, tenant, landlords) is essential.

Our qualitative work in Kimisagara and Gatsata yielded a rich data set. This article draws primarily on eight focus group discussions conducted with owner occupiers, eight focus group discussions with tenants (half female and half male) who were the head of households but excluded local leaders and resident landlords; four focus group discussions with landlords (two landlords-residents, two landlords-absent); two focus group discussions with community health workers; two focus group discussions with village leaders; two class discussions with primary six pupils; three individual interviews with people with disability; three individual interviews with old people (above the age of 65); one person with a chronic disease. 

Survey data were coded and analysed using the SPSS software version 20. For the statistical analysis, the minimum level of significance accepted was 95% (*p* < 0.05). The data collected through focus groups discussions and in-depth interviews with key informants was transcribed and analysed thematically. In order to improve the validity of the data, a triangulation strategy was used. This strategy involved collecting information from a range of sources (household survey, transect walks, focus group discussions, interviews).This has the advantage of filling weaknesses or gaps in data for one method, which results in strengthening the overall quality of the results. Ethical approval was given by Ethics Committee of University of Surrey. Participation in this study was voluntary and all respondents gave verbal informed consent to their participation in the research.

## 3. Results

### 3.1. Socio-Economic Characteristics of Survey Respondents

The age of respondents ranged from 18 years to 88 with a mean of 33.1 years and a SD of 11.7. The majority of the respondents were between the ages of 25 and 35 years. 62.8% of respondents were married with 23.1% being single and 14.2% being divorced/widowed. Men were more likely to be single (35.5%) than female (16.8%) while the women were more likely to be widowed (19.8%) than men (3.1%). Just over half of the respondents had completed primary school education (53.3%). Women were more likely to have had no education than men (11.7% compared to 6.4%) while men were likely to have higher education than women (3.2% compared to 1.4%). 

The level of unemployment was 22.5% while employment was reported to be 77.5%. Of those who said they were employed, the majority were self-employed (66.2%) including farm work, 20.6% were engaged in waged employment, while 13.2% of respondents were dependent family workers. Men were more likely to be in waged employment (25.5%) than women (9.3%) while men were significantly (*p* < 0.005) less likely to be unemployed (11.3%) than women (30.2%) (Cramer’s V < 0.005). During the focus group discussions with males and females in the two selected settlements, it was consistently revealed that men were financially responsible for the family, although women may partake in small income-generating activities. In terms of household duties, men were mainly responsible for providing food, shelter, clothing, construction of latrine; whereas, women were mainly engaged in domestic work, childcare, raising the children, cooking, cleaning, and collecting water. 

### 3.2. Existing Sanitation Systems in Study Areas of Kigali

In the two informal settlements in Kigali, pit latrine facilities (both with a slab and without a slab) were the most common sanitation option (91.2%), as shown in Table 1, Section (a). The table also shows the distribution of improved sanitation systems according to WHO/UNICEF Joint Monitoring Programme (JMP) definition, which excludes shared sanitation. Improved sanitation facilities are defined as the hygienic separation of human excreta from human contact, which includes a flush or pour flush toilet connected to either pipe sewerage, a septic tank or a pit latrine, a Ventilated Improved Pit latrine (VIP), a composting toilet, a pit latrine with a cover slab and other special case (e.g., urine diverting dry toilet) [5]. Thus, when shared sanitation is excluded from improved sanitation, the proportion of residents with improved sanitation in study areas is 18.4% (Table 1), Section (b). 

**Table 1:** Distribution of improved sanitation systems (a) and shared usage according to JMP definition (b) in informal settlements of Gatsata and Kimisagara.

	Section (a): Type of Sanitation Systems			Section (b): Sanitation Systems					
				Shared		Not Shared		Grand Total	
		N	%	N	%	N	%	N	%
**Improved**	Flush	48	2.7	19	1	29	1.6	48	2.6
	Pit latrine with slab	946	52.7	650	36.3	296	16.50	946	52.9
	Other improved categories	15	0.9	11	0.6	4	0.3	15	0.9
**Improved Sub-Total**		1,009	56.3 **	680	37.9	329	18.4 *	1,009	56.3
**Unimproved**	Open pit latrine without slab	690	38.5	558	31.2	132	7.4	690	38.6
	Other unimproved categories	90	5.0	74	4.1	16	0.9	90	5.0
	Open defecation	5	0.3	-	-	-	-	5	0.3
**Unimproved Sub-Total**		785	43.7	632	35.3	148	8.3	785	43.6
**Grand Total of both Improved and Unimproved**		**1,794**	**100**	1,312	73.2	477	26.7	1,794	100

Notes: * The proportion improved sanitation (Shared systems excluded); ** the proportion improved sanitation (Shared systems not excluded).

Sharing facilities was common with, on average a latrine, being shared, between four households. At the extreme, in one instance, 15 households were sharing one latrine. Open defecation was not widely reported (0.3%) (Table 1), Section (a) despite being observed during the transect walks. The few households that were not using latrines gave a number of reasons, that the latrine was full; they did not have a latrine; the latrine had collapsed; latrine was under construction. 

### 3.3. Issues Associated with Existing Sanitation Facilities in Informal Settlements of Kigali

**Figure 1:**
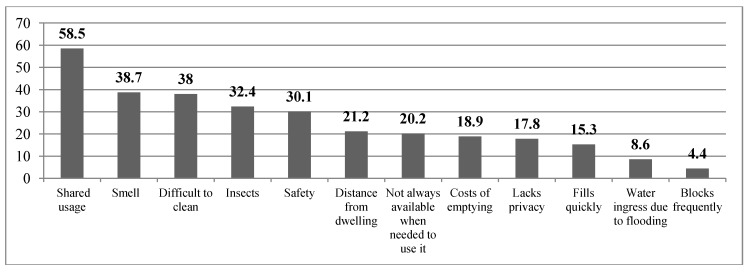
Problems with existing sanitation systems in informal settlements of Kigali as reported by respondents.

The survey sought to establish whether respondents think there are problems associated with their sanitation facility. 80% of respondents reported at least one problem, with the most frequently mentioned problem being shared usage (58.5%) followed by smell (38.7%). Other problems frequently reported were difficulty to clean (38%), insect problems (32.4%), safety (30.1%), distance from the dwelling (21.2%), and toilet not always available when needed (20.2%) (Figure 1). 

However, while some respondents experienced no problems, others said that they had more than one problem with a majority of respondents having between two and five problems (20.8%, 16.9%, 13.4%, and 8.5% respectively). A few households reported experiencing more than six problems (5.5%). During transect walks, we observed that most of the latrines were open pits that smelled bad because they were very shallow or full; a few had visible breeding areas for flies. The mud floors in traditional pit latrines had dirty floors, preventing the water draining away in a hygienic sanitary way and providing a favourable breeding ground for flies. 

The participants in focus group discussions said that most of toilets were dirty and this caused health problems, such as intestinal worms, typhoid and diarrhoea, especially amongst children. One female tenant, for example, explained how diseases can be contracted from using unclean toilets:

*“The ways of transmission are various. The lack of fresh water and soap in the house can hinder people from hand washing and uncovered pits or stagnant black water can attract flies. Effluent from tanks and pits can pollute surface and ground water used for human consumption with pathogens and pollutants.”*

One reason for the unhygienic condition of the toilets was said to be because a large number of households share one latrine. For example, elderly, disabled and sick people reported unclean facilities to be a big challenge for them, especially when shared with many households. This was because they sometimes had to touch the floor or dirty walls to get support while using the facility particularly when squatting in position caused pain in their knees and back. The following quote illustrates their concerns:

*“Sometimes when I stay too long in the squatting position, to be able to stand up, I find support by touching the ground, where I can easily contaminate with diseases. It is a very embarrassing and stressful experience for us old people” (Woman with disability).*

The lack of lighting in the toilets was frequently mentioned by tenants; however, only a few owner occupiers also raised this issue. Some tenants noted that the lack of lights caused people to not see the toilet hole at night and thus to defecate on top of the toilet. While a lack of doors was cited by the majority of tenants, owner occupiers expressed concern about the toilets that cannot be locked while occupied in, creating a fear of the sexual assault on the part of women. Tenants and owner occupiers complained of toilets that smell bad in the house and flies from the toilet getting into the kitchen, which spreads diseases:

*“When you are passing by, the bad smell from the toilets hit your face and you do your best to leave the place as quick as possible” (One male tenant).*

Disabled people with mobility problems have different views about the distance to toilets, and said that the toilets were too far away from their house. A related issue was that the paths to the toilets were not easy for disabled or old people to use because they are not paved. One disabled woman explained:

*“When constructing the toilets and paths to the toilet, they do not give any considerations to the disabled; everything is constructed to suit the ordinary normal person”.*

Another disabled man, who uses his hands to walk, discussed his problems getting to the toilet:

*“The path to the latrine is not safe at night. I fear I will get injured by stones or sharp objects left on my way. Thus I am obliged to defecate beside the house like a kid and my wife has to wipe it off early in the morning”.*

Some toilets were reported to be constructed with poor materials such as timber and sticks; thus people feared using them as they could be prone to accidents, such as falling into the pit. Although only one owner occupier mentioned a fear of slipping and falling on the wet toilet floor, the issue of safety was frequently raised by tenants.

*“I am frightened to use our toilets because I fear that the toilets will collapse at any time and I will fall downer the hole and die; so I prefer other alternatives such as using a bucket, or practicing open defecation” (Male tenant).*

They further pointed out that some toilets lacked doors and this compromised privacy:

*“My toilet is okay, the only problem is that it is not properly constructed and does not promote privacy. If I am inside, people outside can see me while using it”, complained one female tenant.*

Older and disabled people frequently mentioned difficulties they have using a toilet without a seat and explained that their knees and legs get tired easily. To deal with such a situation, they said that they made a kind of stool for them to sit on to go over and around the pit hole. They also said that they used buckets in their homes and their relatives throw out the waste later.

Tenants reported that the size of the holes in some pit latrines are too large for children, thus they defecate on the ground or besides the pit latrine’s hole. They also complained about problems with open defecation practiced by people without toilets in their settlement. Similarly, owner occupiers reported the issue of open defecation when their pits fill up:

*“People in settlements still practiced open defecation in open spaces between houses, in corners, on people’s fences and this is mostly done by kids, drunkards, homeless youth or outsiders; the reasons of such malpractice was said to be due to local bars not having a toilet as well as lack of public toilets in the settlement, unavailability of toilet due to a large number of people sharing a facility, poor hygienic conditions of the facility, people without facilities or simply because of poor attitude of some people” (Female owner occupier).*

Although open defecation was frequently cited by tenants and owner occupiers, village leaders went further and said that people defecate in bags and throw the bags on the roofs. In addition, they reported that people empty the toilets into the drainage channels and solid waste dumps at night or in neighbours’ toilets. However, observations made during the transect walks found that there was limited open defecation and only few cases were reported in the backyards of house. 

The tenants also complained about the pits, which fill quickly. One female tenant stated:

*“Pit latrines fill up quickly here in informal settlements as a result of the large number of users. Due to the diminishing space available in informal settlements, households must resort to emptying their pit latrine and as constructing a new latrine is impractical, but the issue is the fact we do not even have emptiers around here...”*

While agreeing to some extent with tenants about the pits that filled quickly, landlords provided a slightly different point of view. They stressed that in most cases; pits had shallow depth due to high water table or rocky soil, and blamed tenants for disposing of tins, glasses, plastics and garbage into the facility, thus causing the facility to fill up quickly. 

### 3.4. Constraints to Sustainable Sanitation in Informal Settlements of Kigali

Results from the survey showed that the most important constraints to building sanitation facilities were lack of money (68.2%), topography (13.8%), insufficient space (12.3%), difficult in obtaining permit (3%), lack of construction materials (2.4%), lack of specialized equipment (0.2%) and lack of information (0.2%). As far as toilet waste emptying and transport were concerned, only 2% of respondents reported emptying their pit latrines. Yet when a pit fills up, emptying is often the only sustainable option [1]. 

**Figure 2:**
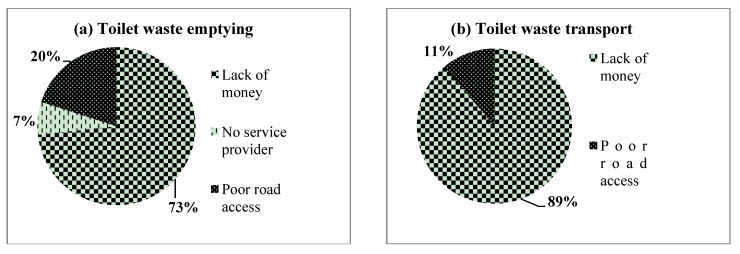
The most important constraints in toilet waste (a) emptying and (b) transport in informal settlements of Kigali.

Of the 25 cases where households emptied their pit latrines, 34% transported the waste to the landfill site at Nduba while the remainder disposed of their waste indiscriminately in dumpsites. This shows that waste emptying and transport services are almost non-existent in informal settlements of Kigali. The survey sought to understand why such a situation exists by ascertaining the most important barriers to emptying and transport (Figure 2). It was found that lack of money was a major constraint (73% for emptying and 89% for transport).

As observed during the transect walks, the informal settlements were constructed on the most undesirable land such as landslide-prone steep slopes, flood plains, swampy grounds, and rocky grounds that are hard to build on. This presents a big challenge in terms of emptying and transporting waste from pit latrines. In addition, during the qualitative research, the landlords’ unwillingness to improve the sanitation facilities was frequently mentioned by tenants as a big constraint:

*“Landlords become resistant when asked for improved sanitation and are more concerned with making money. For instance, when tenants request for improved sanitation landlords respond harshly telling them to look for other houses where they will find what they want and if they are not satisfied with what is there, that there are many people who can be comfortable with what is present” (Female tenant).*

However, the views of landlords were different from those of tenants. While the tenants said that landlords were unwilling to spend money to improve sanitation, landlords explained that they were not planning to upgrade sanitation facilities on their properties because they did not receive enough money from rent and therefore they could not afford to improve sanitation. These views from landlords were shared by a few tenants who noted that some landlords might be willing to pay for improved sanitation but might themselves face financial constraints. A female tenant said:

*“Sometimes, we think that landlords are rich and yet they are not, and when you analyse you find that they cannot afford to construct a good standard toilet, because the landlords have also many dependents and more pressing needs and priorities than having a proper toilet”.*

Owner occupiers shared some of views of tenants and landlords and stated that sanitation is not their top priority because they had many other urgent issues to deal with:

*“My toilet has no roof because I have no money; when I get money, another urgent problem occurs and then I prefer to solve that one first” (A female owner-occupier).*

The participants in the qualitative research also raised the challenges in achieving sustainable sanitation in informal settlements of Kigali. For example, the landlords noted that when an old pit is full, they dig a new hole because emptying services are limited and expensive:

*“The major challenge faced is the cost of constructing a toilet which is very expensive. Even if they pay us rent every month, it is too little to construct a toilet” (Male resident- landlord);*

*“Space is the biggest obstacle of all; it is impossible to construct more than three toilets on my land” (Female absentee-landlord).*

Owner occupiers agreed that although the lack of money was one of reasons why emptying services were limited in informal settlements of Kigali, there were other constraints that needed to be taken into consideration:

*“Although pit emptying services are too expensive (USD 150) and few can afford to pay this amount of money, they (emptying services) had been banned by the authorities because people had dumped the sewage into the drainage channels; yet those who afford to pay for emptying are limited by the roads which are too narrow in the settlement for pit emptying trucks to reach their toilets, so I think the facilities that are in close proximity to roads could be easily emptied” (A male owner-occupier).*

Some key informants went further and pointed out that there was nowhere in Kigali for toilet emptiers to dump the sewage from the toilets:

*“We really do not have sustainable mechanism to deal with sewage here because there is nowhere to dump the sewage; it is a big problem...”(KCC official).*

Responses for elderly, disabled and sick people showed that while flush toilets suited their needs, the lack of piped water to their compounds makes it impossible for them to have flush toilets:

*“The facility that I think is suitable for older people is the flush toilet as it gives support but the challenge is there is no water access in the settlement and this would also increase water bills yet money is one of my biggest challenges in accessing a good toilet” (Old woman).*

## 4. Discussion

The aim of this study was to analyse challenges to achieving sustainable sanitation in informal settlements in Kigali and frame appropriate, suitable and sustainable sanitation technologies that match with the local conditions of study settlements of Kigali. 

### 4.1. Challenges to Achieving Sustainable Sanitation in Informal Settlements of Kigali

Regarding the challenges to achieving sustainable sanitation in informal settlements of Kigali, it was found that the high population density and the ensuing congestion of houses contribute to the lack of space for latrines. This leads to pits being dug close to houses, which weaken the foundations of already poorly constructed houses. The majority of the informal low-income communities were located in difficult terrains such as marshy land, swamp, steep slopes, abandoned refuse tips or grave yards, flood plains and rocky areas. Building latrines in these soil conditions can sometimes be challenging due to the instability of the soil, and the difficulty of digging into rocks.

Pit latrines with a slab represent improved sanitation in its most basic form, but once the pit is full it no longer provides this service; and the pit must either be covered over, and a new latrine constructed, or the existing pit emptied [18,19]. However, unlike other developing cities such as Kampala, Nairobi and Dar-es-Salaam, Kigali has no clear strategy for the emptying of pit latrines [1,15] and only 2% of households empty sludge from their pits. Therefore, there is a risk of the full latrine overflowing, contaminating the environment with large quantities of excreta containing harmful pathogens and causing offensive smells [20].

However, smell was not reported as a major problem in some areas of Africa [21,22]. From the findings of this study, a possible explanation for this disparity could be that, a significant proportion of pit latrines in the informal settlements of Kigali might be simple pit latrines without a cover and are thus technologically inferior at reducing smell when compared to VIPs.

Another problem that was noted in relation to full pits is the potential for the pollution of groundwater under or near pit latrines, particularly in areas with high water table [23], which is the case of most of informal settlements of Kigali. This is a serious problem because it affects the quality of drinking-water. For example, due to the high water table, Botswana experienced high groundwater pollution that can be linked to the widespread use of pit latrines as a sanitation option [22,24]. 

Pits in the informal settlements of Kigali were generally not lined with bricks and vulnerable to collapse. This puts children at risk and most households tend to discourage children from using latrines for fear that they might fall in. This was also reported in Kumasi, Ghana where children were made to defecate in plastic containers, which were later emptied into the latrine [25]. 

The other issues raised by respondents included closeness of the toilet to the households, accessibility during night, toilet rooms not having light inside, a toilet that is not lockable for privacy when in use, and the propagation of flies. However, although the quantitative results from the survey indicate low percentages for insect nuisance (32.4%), flies and insects are serious issues because they are reported to be responsible for the propagation of faecal-oral diseases, such as diarrhoea or intestinal worms [26,27], children are particularly known to be more at risk [28] because they are used to play in stagnant wastewater. Controlling smells, flies and mosquitoes is, therefore, a high priority for reducing household and environmental health hazards.

A number of factors have constrained progress towards sanitation improvement. In particular, lack of money was reported in survey to be the main reason. The inability to save funds to invest in longer-term sanitation facilities, coupled with a low income, significantly restricts the choices that individuals can make. The situation might be improved if financial support from local and national governments was available. However, unlike other developing cities, there are no Non-Government Organizations (NGOs) working on sanitation issues in the informal settlements of Kigali [15]. In addition, sanitation is not a budgetary priority for the Government [29] and the City of Kigali, which is supposed to support the poor, often lacks financing resources to meet the needs of the population for sanitation and other services [15]. The illegal nature of occupancy and the impact that is likely to have on decisions to invest in sanitation is another challenge. 

Research in South Africa supports the argument that poverty is the biggest factor in preventing households from benefiting from improved sanitation [30]. However, there was no agreement amongst the participants of this study as to whether financial problems are *a real* or rather *a perceived constraint* that restricted poor urban dwellers of informal settlements from building latrines. Indeed, the results from the questionnaire survey indicated that there were many other factors that hinder the sanitation improvement in informal settlements of Kigali such as the lack of sufficient space to build individual toilets. This is supported by a study conducted in Kibera slum in Nairobi (Kenya) where it was found that it is not feasible to provide individual sanitation facilities in high-density slums with high poverty levels [6].

### 4.2. Sustainable Sanitation Technologies Appropriate to Informal Settlements of Kigali

In developing countries, unimproved sanitation facilities are the prime cause of widespread and serious health problems, but improvements in these services show few health benefits unless they are coupled to improved hygiene behaviour [18]. Overall, 18.4% of households in the informal settlements surveyed had access to some form of improved sanitation system if WHO/UNICEF Joint Monitoring Programme (JMP) definition is used, which excludes shared sanitation. However, this does not genuinely reflect the reality on the ground. This is because access to improved sanitation systems was assessed using physical measures [19] but in most cases this did not capture real access levels since even the flush toilets might not provide adequate sanitation services if they are poorly maintained.

Some respondents argued that the establishment of central sewerage systems can help in addressing some of the challenges and achieving sustainable sanitation in Kigali. However, such systems need high investment and most developing cities, including Kigali, lack the financial resources to pay for centralized sanitation systems [1]. Costly centralized sanitation systems are not only a problem for developing countries. Because of high maintenance cost and little profit returns, centralized or off-site water and sanitation systems have to be directly cross subsidized and the chances to ever become financially sustainable are low even in developed countries [1]. There are other problems caused by centralized sanitation systems associated with over exploitation of natural resources. To transport human waste, networks of sewer pipes consume enormous volumes of water, which is not available in informal settlements.

In comparison with central sewerage systems, decentralized sanitation systems have been promoted by scholars [1,2]. Decentralized sanitation technologies used worldwide include simple pit or traditional latrines, Ventilated Improved Latrines (VIP), Ecological (Eco-san) latrines, pour-flush latrines and Water closet toilets, connected to septic tank. In household survey, it was reported that the majority of respondents reported the use of traditional pit latrines. The preponderance of traditional pit latrines is in line with previous research findings, in which they are reported to be the preferred sanitation option compared to other more advanced technologies such as flush toilets [31] because they are cost-effective and when well designed, built and maintained, they provide adequate sanitary benefits. 

However, the problem is that the pit is not lined and thus difficult to empty when they fill up [32]. The unlined pits pose a challenge using emptying trucks because of the excreta mixing up with soil or gravel particles from the pit walls. A common problem with unlined pit latrines is collapse, especially during the rainy season. Excavating new pits within an ever diminishing space due to the high density and close proximity of households is not practicable or sustainable. Other inconvenient drawbacks for traditional pits are soil and ground water contamination with pathogens, bad odours, flies/mosquito breeding, potential pit collapse in cases of heavy rains, the distance from house, especially for women and children during night [33]. 

On the other side, the flushing toilets connected to septic tanks which constitute the preferred system for most citizens because of their comfort, are also faced with problems. Septic tanks are expensive and therefore not affordable for the majority of the urban poor population and they cease to work properly when they are old and over-utilized, potentially causing serious environmental and public health problems [1]. However, there is no need to start from nothing, since small-scale household composting and other decentralized systems (DeSaR) are widely considered a potential solution for developing countries [2]. Unlike conventional sewers which require a large wastewater system and expensive treatment before there can be any reuse and redistribution, decentralised systems are cost-effective and enable a more efficient separation of liquid and solids for re-use by communities located near the site [1,2]. 

## 5. Conclusions

The aim of this study was to analyse challenges to achieving sustainable sanitation in informal settlements of Kigali and propose sustainable sanitation technologies that match with the requirements of study settlements of Kigali. The study used a mixed method approach and this included transect walks, a household survey, focus groups discussions and key informant interviews. 

Our findings reveal that the traditional pit latrines are the most common types of excreta management systems that exist in Kigali. However, such systems are not a sustainable sanitation option because they are vulnerable to leakages, collapse during heavy rains and attract flies. In addition, these facilities fill up quickly due to small volumetric capacity for most pits, high number of users (because of sharing), and are not easily or regularly emptied. Also, as a result of the steady increase in the population of the slum dwellers coupled to the construction of unplanned structures, the space available for constructing new traditional pit latrines is continually decreasing [34].

To this end, this implies that dwellers of informal settlements are inclined over time to reject these traditional pit latrines for alternative low-cost more sustainable options, such as innovative decentralized *sanitation* and reuse (DeSaR) and water serving sanitation technologies, because they can play a part in reduction of over exploitation of natural water sources, which continue to be scarce, as a result of population pressure in the country. DeSaR technologies are appropriate in informal settlements because they occupy less space, do not require emptying by vacuum tankers, pre-treatment/composting, provides opportunity for nutrients re-cycling which is environmentally sustainable and, if well maintained, have minimal harmful effects [35]. However, to be able to provide improved sanitation options for these communities, pilot projects are necessary so as to gauge acceptability [34]. Meanwhile, since the majority of residents do still depend on shared sanitation facilities to reduce the sanitary-related diseases, more emphasis has to be placed on hygiene education practices, focusing on proper use and cleanliness of the facilities. 

## References

[1] Sano J.C. (2007). Urban Environmental Infrastructure in Kigali City, Rwanda: Challenges and Opportunities for Modernised Decentralised Sanitation Systems in Poor Neighbourhoods. M. Sc. Thesis.

[2] Oosterveer P., Spaargaren G. (2010). Meeting Social Challenges in Developing Sustainable Environmental Infrastructures in East African Cities. Social Perspectives on the Sanitation Challenge.

[3] Shah N. (2012). Characterizing Slums and Slum-Dwellers: Exploring Household-level Indonesian Data. http://storage.globalcitizen.net/data/topic/knowledge/uploads/20120920133613196030_slums_Shah.pdf.

[4] Dinye R.D., Acheampong E.O. (2013). Challenges of slum dwellers in Ghana: The case study of Ayigya, Kumasi. Mod. Soc. Sci. J..

[5] WHO/UNICEF (2010). Progress on Sanitation and Drinking-Water: 2010 Update.

[6] Schouten M., Mathenge R. (2010). Communal sanitation alternatives for slums: A case study of Kibera, Kenya. Phys. Chem. Earth..

[7] National Institute of Statistics for Rwanda (2012). 2012 Population and Housing Census: Provisional Results.

[8] National Institute of Statistics of Rwanda (2010). Rwanda Demographic and Health Survey 2010: Final Report.

[9] Hohne A. State and Drivers of Change of Kigali’s Sanitation—A Demand Perspective. Proceedings of the East Africa Practitioners Workshop on Pro Poor Urban Sanitation and Hygiene.

[10] Parkinson J. (2008). Improving servicing of on-site sanitation-a neglected issue for the UN Year of Sanitation. Water.

[11] Lubaale G.N., Musyoki S.M. Pro-poor Sanitation and Hygiene in East Africa: Turning Challenges to Opportunities. Proceedings of the East Africa Practitioners Workshop on Pro Poor Urban Sanitation and Hygiene.

[12] Scheinberg A., Spies S., Simpson M.H., Mol A.P.J. (2011). Assessing urban recycling in low-and middle-income countries: Building on modernised mixtures. Habitat Int..

[13] Satterthwaite D. (2004). The Under-Estimation of Urban Poverty in Low and Middle-Income Nations.

[14] Tilley E., Morel A., Zurbru C., Schertenleib R. (2008). Compendium of Sanitation Systems and Technologies.

[15] Tsinda A., Abbott P. (2012). A Review and Analysis of the Situation Pertaining to the Provision of Sanitation to Low-Income Settlements in Kigali City (Rwanda).

[16] Chevalier J.M., Buckles D.J. (2013). Participatory Action Research. Theory and Methods for Engaged Inquiry.

[17] Lüthi C., Panesar A., Schütze T. (2011). Sustainable Sanitation in Cities: A Framework for Action, Sustainable Sanitation Alliance (SuSanA) & International Forum on Urbanism (IFoU).

[18] Grimason A.M., Davison K., Tembo K.C., Jabu G.C., Jackson M.H. (2000). Problems associated with the use of pit latrines in Blantyre, Republic of Malawi. J. R. Soc. Promote. Health.

[19] Mtungila J., Chipofya V. (2009). Issues and challenges of providing adequate sanitation to people living on the shore of lake Malawi: Case of Monkey Bay, Malawi. Desalination.

[20] Moe C.L., Rheingans R.D. (2006). Global challenges in water, sanitation and health. J. Water Health.

[21] Saywell D., Shaw R. (2005). On-plot Sanitation in Urban Areas.

[22] Bolaane B., Ikgopoleng H. (2011). Towards improved sanitation: Constraints and opportunities in accessing waterborne sewerage in major villages of Botswana. Habitat Int..

[23] Cross P., Morel A. (2005). Pro-poor strategies for urban water supply and sanitation services delivery in Africa. Water Sci. Technol.: J. Int. Assoc. Water Pollut. Res..

[24] UN-HABITAT (2010). UN-Habitat Annual Report 2009.

[25] Adubofour K., Obiri-Danso K., Quansah C. (2013). Sanitation survey of two urban slum Muslim communities in the Kumasi metropolis, Ghana. Environ. Urban..

[26] Prüss A., Mariotti S.P. (2000). Preventing trachoma through environmental sanitation: A review of the evidence base. Bull. World Health Organ..

[27] Graczyk T.K., Knight R., Tamang L. (2005). Mechanical transmission of human protozoan parasites by insects. Clin. Microbiol. Rev..

[28] Thye Y.P., Templeton M.R., Ali M. (2011). A critical review of technologies for pit latrine emptying in developing countries. Crit. Rev. Environ. Sci. Technol..

[29] Tremolet S., Kolsky P., Perez E. (2010). Financing On-Site Sanitation for the Poor: A Six Country Comparative Review and Analysis. Water and sanitation Programme. http://www.wsp.org/sites/wsp.org/files/publications/financing_analysis.pdf.

[30] Chinyama A., Chipato P., Mangore E. (2012). Sustainable sanitation systems for low income urban areas-A case of the city of Bulawayo, Zimbabwe. Phys. Chem. Earth..

[31] Kulabako R.N., Nalubega M., Wozei E., Thunvik R. (2010). Environmental health practices, constraints and possible interventions in peri-urban settlements in developing countries—A review of Kampala, Uganda. Int. J. Environ. Health Res..

[32] Isunju J.B., Schwartz K., Schouten M.A., Johnson W.P., van Dijk M.P. (2011). Socio-economic aspects of improved sanitation in slums: A review. Public Health.

[33] Mara D., Alabaster G. (2008). A new paradigm for low-cost urban water supplies and sanitation in developing countries. Water Policy.

[34] Thye Y.P., Templeton M.R., Ali M. (2009). Pit Latrine Emptying: Technologies, Challenges and Solutions. http://www.ewb-uk.org/system/files/Yoke+Thye+report.pdf.

[35] Jha P. (2003). Health and social benefits from improving community hygiene and sanitation: An Indian experience. Int. J. Environ. Health Res..

